# Supplementary material for: Saccharomyces boulardii (CNCM I-745) improves intestinal damage in sepsis by remodeling intestinal flora structure
Source: Front Cell Infect Microbiol. 2025 Jul 31;15:1602792. doi: 10.3389/fcimb.2025.1602792 (PMC12350329; doi:10.3389/fcimb.2025.1602792)
Supplement: Supplementary file 1 [file DataSheet1.zip › summary/summary/7_Differential_analysis/2_LEfSe_result/Group_DW4_vs_DW0_vs_CW4_vs_CW0_vs_BW4_vs_BW0_vs_AW4_vs_AW0_0.05_3.0/LEfSe_Group_DW4_vs_DW0_vs_CW4_vs_CW0_vs_BW4_vs_BW0_vs_AW4_vs_AW0.s.barplot.pdf]

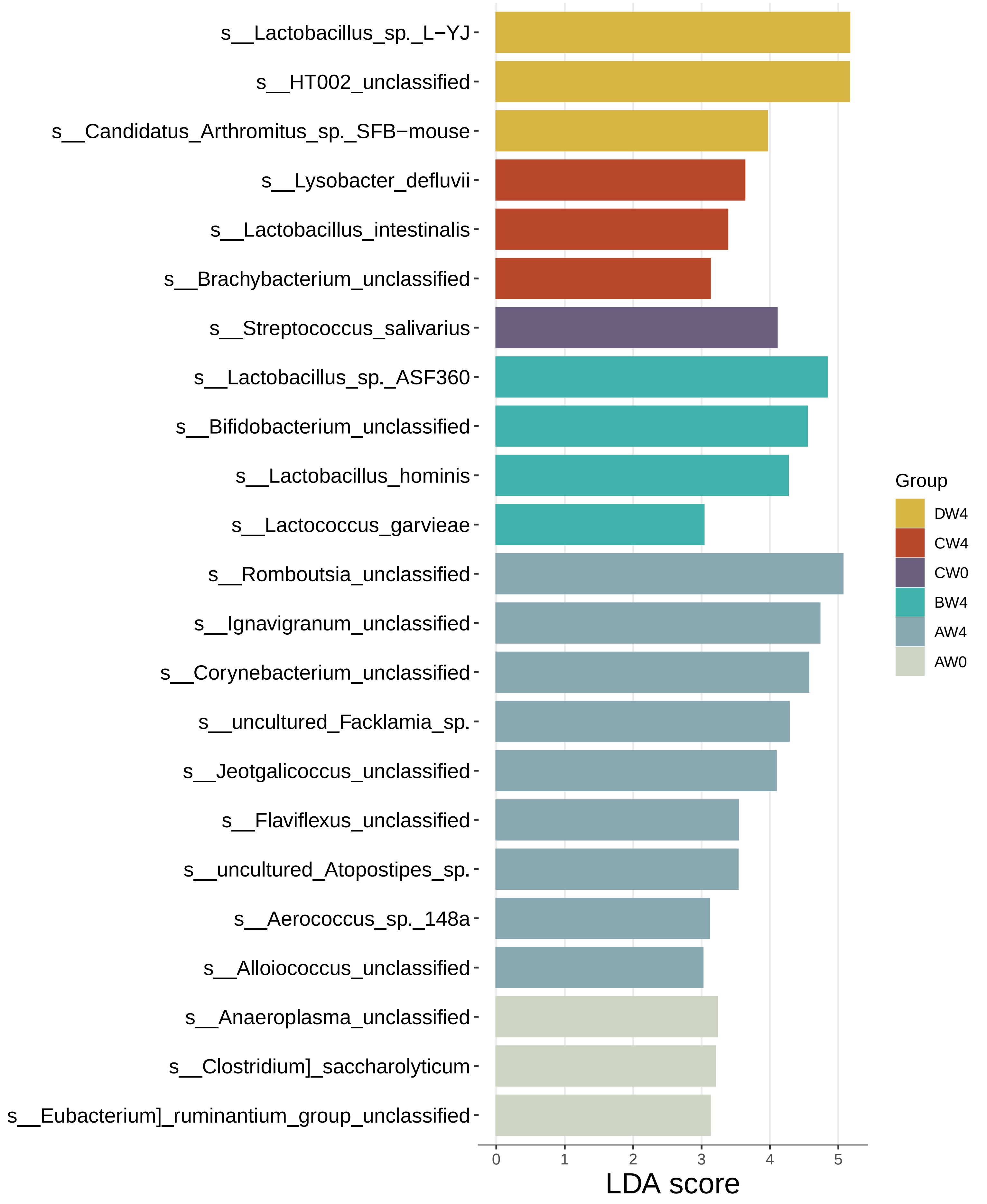
s__Lactobacillus_sp._L−YJ
s__HT002_unclassified
s__Candidatus_Arthromitus_sp._SFB−mouse
s__Lysobacter_defluvii
s__Lactobacillus_intestinalis
s__Brachybacterium_unclassified
s__Streptococcus_salivarius
s__Lactobacillus_sp._ASF360
s__Bifidobacterium_unclassified
s__Lactobacillus_hominis
s__Lactococcus_garvieae
s__Romboutsia_unclassified
s__Ignavigranum_unclassified
s__Corynebacterium_unclassified
s__uncultured_Facklamia_sp.
s__Jeotgalicoccus_unclassified
s__Flaviflexus_unclassified
s__uncultured_Atopostipes_sp.
s__Aerococcus_sp._148a
s__Alloiococcus_unclassified
s__Anaeroplasma_unclassified
s__Clostridium]_saccharolyticum
s__Eubacterium]_ruminantium_group_unclassified
0
1
2
3
4
5
LDA score
Group
DW4
CW4
CW0
BW4
AW4
AW0
